# Circular RNA Expression Profiles and the Pro-tumorigenic Function of CircRNA_10156 in Hepatitis B Virus-Related Liver Cancer

**DOI:** 10.7150/ijms.45637

**Published:** 2020-05-30

**Authors:** Man Wang, Bianli Gu, Guoliang Yao, Peifeng Li, Kun Wang

**Affiliations:** 1 Institute for Translational Medicine, Qingdao University, Qingdao 266021, China.; 2 Henan Key Laboratory of Cancer Epigenetics, The First Affiliated Hospital and College of Clinical Medicine of Henan University of Science and Technology, Luoyang 471003, China.; 3 Department of General Surgery, The First Affiliated Hospital of Henan University of Science and Technology, Luoyang 471003, China.

**Keywords:** Circular RNA, Liver cancer, High-throughput sequencing, Therapeutic target, Cancer biomarker

## Abstract

Liver cancer is one of the most common malignant tumors in the world. Circular RNAs (circRNAs) perform important functions in cancer progression and are regarded as prospective biomarkers for cancer diagnosis and therapy. Here, we used the high-throughput RNA sequencing technology in conjunction with bioinformatics tools to profile circRNA expression in patients with HBV-related liver cancer. A total of 13,124 circRNAs were identified in HBV-related liver cancer, approximately 86.25% of which were sense-overlapping circRNAs. Moreover, 2,996 circRNAs exhibited different expression patterns between liver cancer tissues and matched pericancerous tissues. Function annotation indicated that these aberrantly expressed circRNAs were primarily engaged in cellular processes and cancer-associated pathways. Notably, the circRNA-miRNA interaction networks showed that 6,020 circRNAs were predicted to target 1,654 miRNAs. Quantitative RT-PCR (qRT-PCR) assay indicated that ten randomly selected circRNAs displayed consistent expression patterns with the sequencing results. We further predicted that circRNA_10156 might work as a molecular sponge of miR-149-3p, which served an important function in tumor development. Consequently, our results demonstrated that depletion of circRNA_10156 upregulated miR-149-3p, reduced Akt1 expression, and suppressed liver cancer cell proliferation. The present study will facilitate the elucidation of biological functions of circRNAs in the progression of HBV-related liver cancer providing prospective biomarkers and therapeutic targets for this disease. Our findings also reveal that circRNA_10156 might represent a promising therapeutic target for liver cancer management.

## Introduction

Circular RNAs (circRNAs) constitute a new group of endogenously expressed non-coding RNAs (ncRNAs), which can be found in multiple species ranging from Archaea to humans 1,2. CircRNAs shape into covalently closed loops which neither have 5' to 3' polarity nor a polyadenylated tail 3. In contrast with linear RNAs, circRNAs are highly stable and resistant to degradation by RNA enzymes 4. CircRNAs are generated by back-splicing of precursor mRNAs (pre-mRNAs) where a downstream 5' splice site (splice donor) is linked to an upstream 3' splice site (splice acceptor) 5,6. CircRNAs are originated from exons, ncRNA loci, antisense transcripts, intronic, untranslated, and intergenic regions 7-9. More importantly, circRNAs possess tissue-dependent and developmental stage-specific expression characteristics 10-12. The widespread existence and specific expression pattern of circRNAs suggest their crucial functions in biological and developmental processes. Nevertheless, the exact functions of circRNAs and molecular mechanisms of their action remain largely unclear.

Owing to the advancement in the high-throughput RNA sequencing technology and bioinformatic tools, the functional role of circRNAs has been gradually disclosed. Accumulating studies have verified that circRNAs work as crucial modulators of gene expression 13,14. This regulatory potency of circRNAs is mainly due to their miRNA sponge activity 15. CircRNAs harbor multiple miRNA binding sites, and can sequester and adsorb miRNAs to counteract miRNA-mediated gene silencing effect 16-18. Moreover, circRNAs have been confirmed to participate in diverse biological processes, such as cell viability, proliferation, apoptosis and differentiation 19-21. Remarkably, some circRNAs were dysregulated in human cancers, including lung cancer, esophageal squamous cell carcinoma and melanoma, and these aberrantly expressed circRNAs were engaged in the initiation and progression of these cancers 22-24. Accordingly, circRNAs may act as pivotal players in carcinogenesis and cancer development. Additionally, circRNAs hold immense promise as potential cancer biomarkers owing to their stable structures 25.

Liver cancer, the most prevalent primary hepatic tumor, has become a principal cause of cancer-associated deaths 26. Because of its high rates of invasiveness and recurrence, at least 1000,000 new cases of liver cancer occur annually 27, and nearly 700,000 liver cancer patients die annually, resulting in the low 5-year survival rate in liver cancer patients 28-30. It has been known that chronic hepatitis B virus (HBV) infection serve as a main driver of liver carcinogenesis in high incidence regions 31. Therefore, screening and identifying therapeutic targets and potential biomarkers for HBV-related liver cancer is of paramount importance. It has been found that circRNAs play regulatory roles in the carcinogenesis and progression of liver cancer by sponging miRNAs or altering gene expression. For instance, circMTO1 suppressed the proliferation and invasion of hepatocellular carcinoma (HCC) cells by sequestering oncogenic miR-9 to elevate p21 expression 32. CircRNA_100338 acted as a miR-141-3p sponge and antagonized the invasive potential in liver cancer cells 33. Similarly, another circRNA, cSMARCA5, was reported to repress the proliferation and migration of HCC cells via upregulating the tumor suppressor TIMP3 by targeting miR-181b-5p and miR-17-3p 34. Therefore, it can be concluded that circRNAs serve vital roles in liver cancer pathogenesis. Nevertheless, our knowledge regarding their connection with the initiation and progression of HBV-related liver cancer is still very limited. A better comprehension of the expressional characteristics and biological function of circRNAs in HBV-related liver cancer would be conducive to elucidating the pathogenic mechanism associated with HBV-related liver cancer and provide potential novel therapeutic targets and prognostic indicators for this disease.

Herein, we comprehensively compared circRNA expression profiles between HBV-related liver cancer tissues and matched pericancerous tissues by using the high-throughput RNA sequencing technology combined with bioinformatics tools. We screened differentially expressed circRNAs and characterized their potential functions. To the best of our knowledge, this study provided the first identification and characterization of circRNAs in HBV-related liver cancer by employing the high-throughput RNA sequencing technology. Moreover, we discovered a target molecule, circRNA_10156, and further validated its regulatory role in liver cancer by series of functional experiments. Collectively, our study not only comprehensively revealed the expression profile of circRNAs in HBV-related liver cancer but also shed light on the functional roles of circRNAs in cancer pathogenesis, which will be helpful to explore useful biomarkers and therapeutic targets for the intervention of HBV-associated liver cancer.

## Materials and Methods

### Clinical specimens

The present study was granted by the Ethics Committee of the Affiliated Hospital of Qingdao University and the First Affiliated Hospital of Henan University of Science and Technology. Moreover, this study was carried out in accordance with the provisions of the Helsinki declaration. All patients provided written informed consent for the publication of any associated data and accompanying images. HBV-positive liver cancer tissues and matched paracancerous tissues from five patients were obtained from the First Affiliated Hospital of Henan University of Science and Technology between July and September 2017. Among them, three pairs of liver cancer tissues and matched pericancerous tissues were selected for illumina sequencing. All tissue specimens were collected in the operating room promptly after resection and were subsequently snap-frozen in liquid nitrogen. Clinical and pathological features of the patients with liver cancer were shown in Table 1.

### CircRNA library construction and illumina sequencing

Total RNAs were isolated from liver cancer tissues and paired adjacent normal tissues using the mirVana miRNA isolation kit (Ambion, Austin, Texas, USA) followed by RNase-free DNase treatment to exclude traces of genomic DNA. The quantity and quality of total RNAs were evaluated using an Agilent Bioanalyzer 2100 system (Agilent Technologies, Santa Clara, CA, USA). Ribosomal RNAs (rRNAs) were removed from the total RNAs using the Epicentre Ribo-zero^TM^ rRNA removal kit (Epicentre, Madison, WI, USA). Then, the rRNA-free RNAs were subjected to RNase R (Epicentre) treatment, and the resultant products were purified using Trizol extraction (Invitrogen, Carlsbad, CA, USA). Consequently, the purified RNAs were used as templates for cDNA library construction according to the protocol for the mRNA-Seq sample preparation kit (Illumina, San Diego, USA). Briefly, purified RNAs were first fragmented and reversely transcribed to double-stranded cDNA. The remaining overhangs were converted into blunt ends using exonuclease and polymerase. After adenylation of 3' ends of cDNA fragments, sequence adaptors were ligated with both ends of fragmented cDNAs. The cDNAs with lengths ranging from 150 to 200 bp were screened for library construction. The libraries were clustered on a cBot Cluster Generation System using TruSeq PE Cluster Kit v3-cBot-HS (Illumina) in accordance with the manufacturer's instructions. Following cluster generation, the paired-end sequencing was conducted on an Illumina Hiseq 2500 platform (Oebiotech, Shanghai, China).

### CircRNA identification

Human genome sequences and gene annotations were downloaded from NCBI databases (https://www.ncbi.nlm.nih.gov/projects/genome/guide/human/index.shtml). The total sequencing amount was more than 10 G for each library. The raw reads were filtered by eliminating adaptor-polluted reads and low quality reads. The Q30 and GC content of the clean data were determined. The clean reads with high quality were mapped to reference genome and transcriptome using the BWA-MEM software. CircRNAs could be identified by CIRI algorithm with genomic annotation from NCBI 35. The CIRI algorithm identified circRNA candidates by a two-step filtering. During the first filtering, CIRI collected the paired chiastic clipping (PCC) signals in the Sequence Alignment Map (SAM) of BWA-MEM. The predominant GT-AG splicing signals were used as the PCC. CIRI scanned the SAM alignment again to cluster extra junction reads missed during the first scan and also executed further filtering to remove false positive junctions.

### Determination of differentially expressed circRNAs

Quantification of circRNA expression was performed using the RPM (reads per million mapped reads) algorithm according to the length of specific circRNA and read counts mapped to this circRNA. DEseq software was used to screen differentially expressed circRNAs 36. P-values for gene expression differences were adjusted by false discovery rate (FDR) using the Benjamini-Hochberg (BH) procedure. CircRNAs with P-value<0.05 and |log_2_ (fold change)|>1 were defined as differentially expressed.

### GO and KEGG annotations of circRNA-host genes

CircRNA-host genes were subjected to Gene Ontology (GO) annotations using the Blast2GO software and mapped to terms in Kyoto Encyclopedia of Genes and Genomes (KEGG) pathway database (http://www.genome.jp/kegg/) using the KOBAS software 37-39. The percentage and number of circRNA-host genes assigned to the sub-categories of three main GO domains (Cell Component, Molecular Function and Biological Process) were calculated by WEGO (http://wego.genomics.org.cn/) 40. The statistical enrichment of circRNA-host genes in the KEGG pathways was further analyzed. A corrected P-value of 0.05 was set as a threshold to determine significant enrichment of circRNA-host genes.

### Construction of circRNA-miRNA interaction networks

The miRanda algorithm was employed to identify putative miRNA binding sites in liver cancer circRNAs 41. Target miRNAs were selected according to conserved seed match sequences between circRNAs and miRNAs. Cytoscape 3.4.0 was used to construct the circRNA-miRNA interaction network 42.

### Quantitative real-time PCR (qRT-PCR)

Total RNAs were extracted from tumor tissues of patients with HBV-associated liver cancer or liver cancer cells. Thereafter, the RNA samples were reversely transcribed into cDNA with random primers. The qRT-PCR assay was performed with SYBR^®^ Premix Ex Taq^TM^ II kit (Takara, Dalian, China) on a CFX96 real-time PCR system (Bio-Rad, Hercules, California, USA). The primer sequences used for qRT-PCR were listed in Table S1. The relative expression of each target was calculated using the 2^-ΔΔCt^ method in triplicate experiments 43. The expression levels of circRNAs and mRNAs were normalized to *GAPDH*, while *U6* was used as the internal reference for miRNAs.

### Cell culture and transfection

The liver cancer cell lines (HepG2 and Huh7) and the normal human hepatic cell line L02 were purchased from the American Type Culture Collection (ATCC). The cells were cultured in Dulbecco's modified Eagle's medium (DEME, Invitrogen) supplemented with 10% fetal bovine serum (FBS, Gibco, Grand Island, NY, USA). Cells were cultured at 37 °C in a humidified atmosphere with 5% CO_2_.

A specific siRNA against circRNA_10156 (si-circRNA_10156, 5'-AGGAAATAACCAACTGGAACA-3') and a negative control siRNA (si-NC, 5'-UUCUCCGAACGUGUCACGUTT-3') were synthesized by GenePharma (Shanghai, China). Liver cancer cells were transfected with 50 nM siRNAs using the Lipofectamine 3000 (Invitrogen). Liver cancer cells were subsequently collected at 48 h after siRNA transfection.

### Cell proliferation assay

Cell proliferation assay was carried out using the Cell Counting Kit-8 (CCK-8; Dojindo, Kumamoto, Japan) based on the manufacturer's instruction. Briefly, transfected cells were seeded at a density of 5×10^3^ cells per well in 96-well plates. After 24 h incubation, 10 μL of CCK-8 solution was supplemented to each well at the indicated time points after transfection. Following incubation for 2 h at 37 °C, the absorbance at 450 nm was measured using a microplate reader (Bio-Rad Laboratories, Hercules, CA, USA).

### Statistical analysis

Statistical analysis was performed using the SPSS 18.0 software (SPSS lnc., Chicago, IL, USA). Differences between two groups were analyzed with Student's t-test. Comparisons among multiple groups were conducted using one-way ANOVA followed by Tukey's post hoc test. A significant difference was defined by a P-value <0.05.

## Results

### General profiles of circRNAs in patients with HBV-related liver cancer

To identify circRNAs in patients with HBV-related liver cancer on the transcriptome-wide level, we extracted RNAs from liver cancer tissues and matched adjacent normal tissues from three patients (biological replicates). Following rRNA depletion and RNase treatment, the residual RNA products were used as templates for library construction. The libraries were sequenced on an Illumina Hiseq 2500 platform, yielding a total of 496.82 million raw reads with a mean length of 150 bp (Table 2). Analyses of sequence quality scores indicated that 96.8 to 97.25% of sequencing data pointed above Q30. The mean GC content of clean reads for liver cancer tissues and matched pericancerous tissues ranged from 54.16% to 57.41%. These results demonstrated that the sequencing quality was high and the sequencing data was reliable.

**Figure 1:**
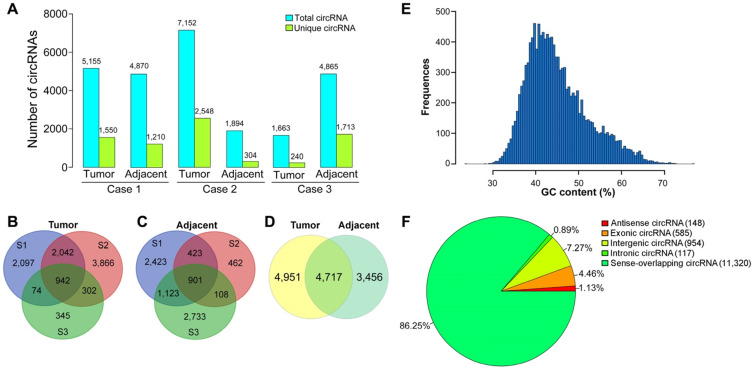
** Identification and classification of circRNAs in patients with HBV-related liver cancer. (A)** The number of circRNAs found in tissues from three patients with HBV-related liver cancer (Cases 1-3). **(B)** Venn diagrams of circRNAs identified in liver cancer tissues from three patients (biological replicates). S1, sample 1, the tissue sample derived from case 1; S2, sample 2, the tissue sample derived from case 2; S3, sample 3, the tissue sample derived from case 3. **(C)** Venn diagrams of circRNAs detected in paired adjacent normal tissues from three patients. **(D)** Venn diagram showing the number of tissue-specifically expressed circRNAs in liver cancer tissues and paired adjacent normal tissues. **(E)** GC distribution of circRNAs. **(F)** Genomic origin of the circRNAs. CircRNAs are divided into five types according to their genomic origin: antisense circRNA, exonic circRNA, intergenic circRNA, intronic circRNA and sense-overlapping circRNA. The number and percentage of circRNAs from different genomic locations are shown.

After filtering low-quality reads and removing the adaptor sequences, 448.58 million clean reads with high quality were then used for circRNA identification. Among the total reads, 68.4, 71.29 and 75.08 million reads were generated from liver cancer tissues of the patients, respectively. Finally, a total of 13,124 unique circRNAs were discovered in the tissue samples by detecting the back-spliced junction reads. Among the identified circRNAs, 5,155 and 4,870 circRNAs were identified in liver cancer tissue and paired paracancerous tissue of case 1, respectively (Figure 1A). In case 2, 7,152 and 1,894 circRNAs were discovered in the tumor sample and adjacent normal tissue. In case 3, 1,663 and 4,865 circRNAs were found in liver cancer tissue and adjacent normal tissue. Moreover, 942 common circRNAs were identified in liver cancer tissues from the three patients (Figure 1B), while 901 circRNAs were co-expressed in the adjacent normal tissues (Figure 1C). We further identified the commonly expressed circRNAs in both liver cancer tissues and the corresponding pericancerous tissues. As shown in Figure 1D, 4,717 common circRNAs were found in both liver cancer tissues and paired pericancerous tissues, and 4,951 and 3,456 were specifically expressed in liver cancer tissues and paired pericancerous tissues, respectively.

To determine the properties of circRNAs in HBV-related liver cancer, the GC content of the circRNAs was depicted in Figure 1E. The GC content of the circRNAs had a single peak spanned from 35% to 45%. It is known that circRNAs are generated by a non-classical form of alternative pre-mRNA splicing known as “back-splicing” 44. CircRNAs are mainly originated from exons, introns, intergenic areas, or the antisense transcripts 45. The origination of the identified circRNAs was predicted via annotation of the circRNAs to the reference genome. It was reported that sense-overlapping circRNAs constituted the majority of circRNAs in other eukaryotic organisms such as zebrafish and trifoliate orange 46,47. Similarly, most of the identified circRNAs (11,320, 86.25%) were generated from sense-overlapping regions (Figure 1F), indicating that the formation of circRNAs is closely associated with pre-mRNA splicing mechanism. Approximately 4.46% (585) and 7.27% (954) of circRNAs arose from exons (exonic circRNA) and the intergenic regions. A small proportion of circRNAs were antisense circRNAs (148, 1.13%) and intronic circRNAs (117, 0.89%).

**Figure 2:**
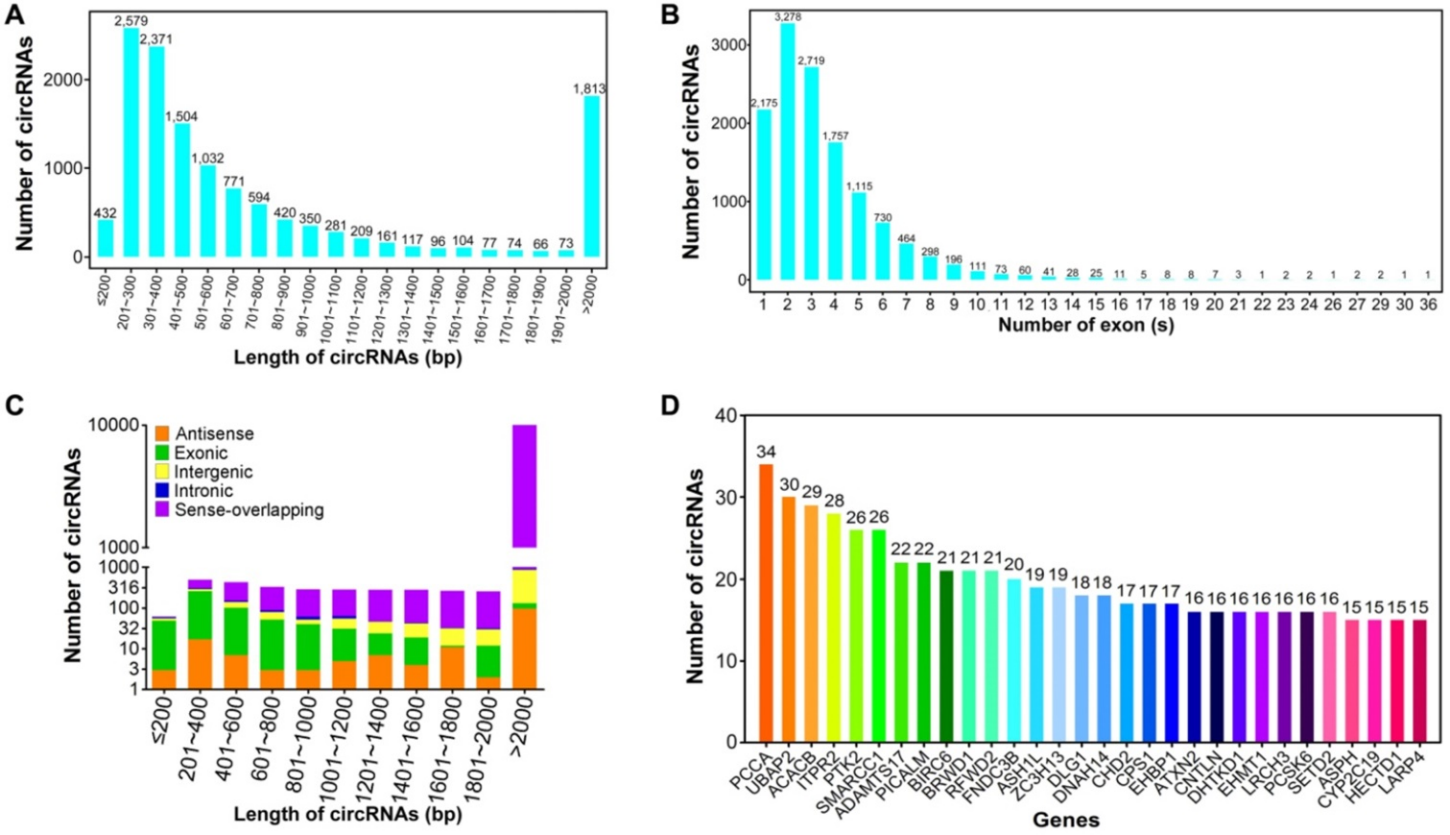
** Sequence characteristics of circRNAs in HBV-related liver cancer. (A)** Length distribution of circRNAs. **(B)** Number of circRNAs that contain varying number of exons. **(C)** Length distribution of circRNAs derived from different genomic locations. **(D)** Number of distinct circRNAs stemmed from each parental gene.

The size of these circRNAs ranged from under 200 bp to over 2,000 bp (Figure 2A). The average length of circRNAs was 2,762.29 bp, most of which (8,851, 67.44%) had a length ranging from 200 to 800 bp. This result was consistent with previous studies of circRNA transcriptomes in human brain, heart and lung 48,49. About 3.29% (432) of circRNAs were predicted to have a length of below 200 bp, wherein 29.27% (3,841) of circRNAs were greater than 800 bp in length. Based on human gene structure annotations, the majority of circRNAs (11,044, 84.15%) were composed of no more than five exons derived from parental genes, whereas only 2.14% (281) of exonic circRNAs had more than ten exons (Figure 2B). The length count analysis indicated that the majority of exon-derived circRNAs (471, 80.51%) were shorter than 1000 bp, whereas most of the intergenic (836, 87.63%) and sense-overlapping circRNAs (10,378, 91.68%) were longer than 1000 bp (Figure 2C). In the eukaryotic genome, the non-coding regions are more and longer than the protein-coding regions 50, leading to the shorter length of exonic circRNAs than intergenic circRNAs. The circRNA-producing genes were further analyzed. A total of 12,170 circRNAs (92.73%) were found to be stemmed from 4,607 unique parental genes. Of these genes, 2,466 circRNA-host genes (53.53%) produced two or more circRNAs, implying that the regulation mechanisms of alternative pre-mRNA circularization in HBV-related liver cancer are complex and need further detailed investigation. Notably, 11 parental genes (0.24%) could produce more than 20 circRNAs (Figure 2D) which remained in agreement with the conclusion that circRNAs are generated by alternative pre-mRNA splicing 51.

### Functional annotation of liver cancer circRNAs

It is well known that the biological function of circRNA is associated with that of their linear isoforms. Functional annotation of circRNA-host genes provides important clues regarding the biological significance of the corresponding circRNAs. GO and KEGG analyses were conducted to predict the functions of circRNA-host genes. GO functional classification was made on three different categories, consisting of cellular component, molecular function and biological process. As a result, 11,724 circRNA were categorized into 18 cellular component terms, 16 molecular function terms, and 34 biological process terms, in total 68 functional terms (Figure S1). In the cellular component aspect, most of circRNA-host genes were located within 'cell' (10,786, 92.0%), 'cell part' (10,779, 91.94%) and 'organelle' (9,646, 82.28%). The main enrichments for the molecular function aspect were 'binding' (10,037, 85.61%) and 'catalytic activity' (4,886, 41.68%). As for the biological process terms, the top three largest categories were 'cellular process' (10,068, 85.88%), 'metabolic process' (8,029, 68.48%) and 'biological regulation' (7,875, 67.17%), indicating that the circRNA-host genes were extensively involved in metabolic activities.

KEGG categories were applied to disclose the involvement of circRNA-host genes in biological pathways, and 4,579 circRNA-host genes were mapped to 295 KEGG pathways. Specifically, the top five KEGG pathways were 'Pathways in cancer' (311, 6.79%), 'Endocytosis' (294, 6.42%), 'Ubiquitin mediated proteolysis' (264, 5.77%), 'Proteoglycans in cancer' (255, 5.57%) and 'Regulation of actin cytoskeleton' (241, 5.26%) (Figure S2).

### The differential expression patterns of circRNAs in HBV-related liver cancer

**Figure 3:**
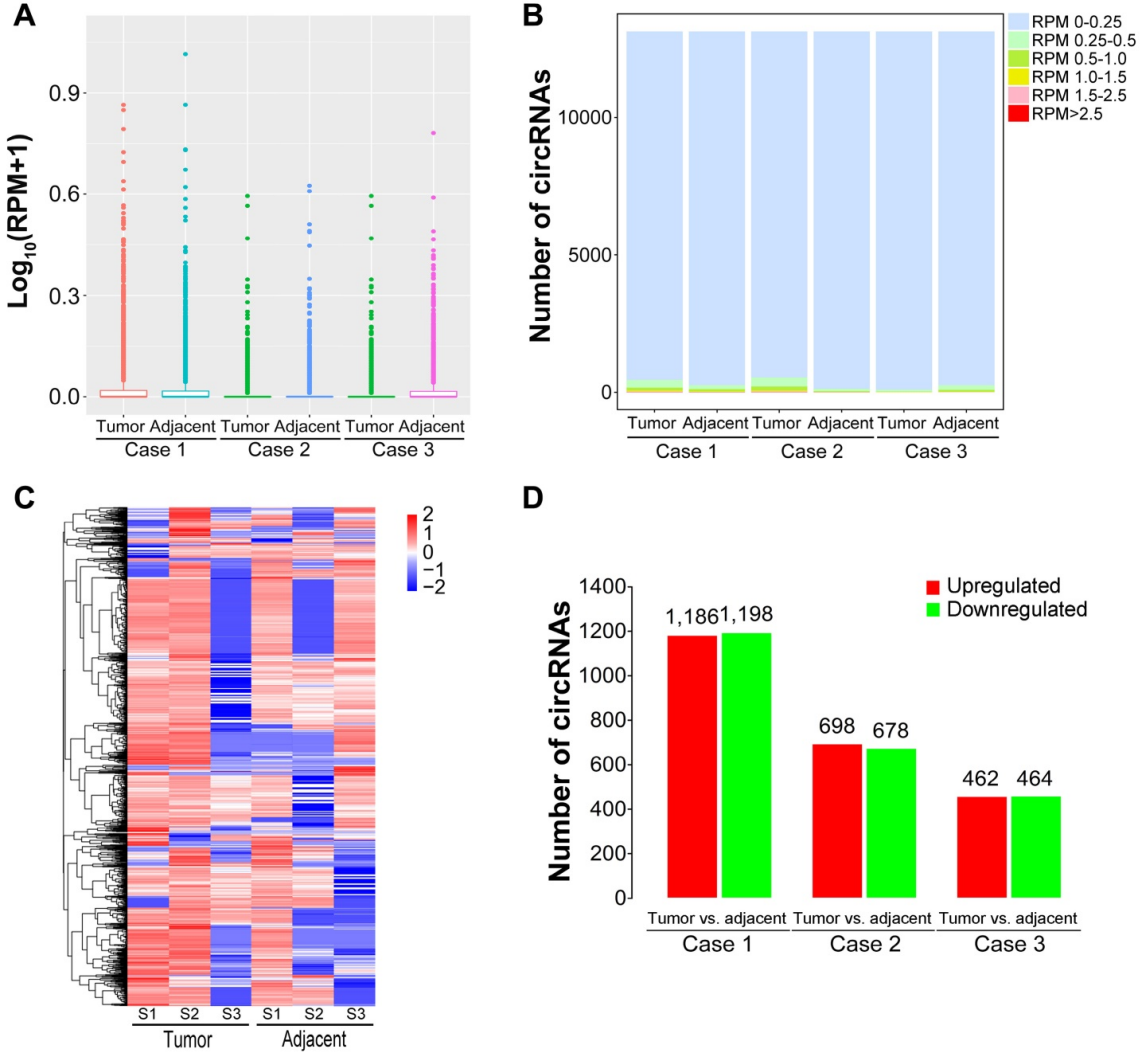
** Expression patterns of circRNAs in HBV-related liver cancer. (A)** Box plots of RPM values of circRNAs in HBV-related liver cancer. **(B)** The distribution of RPM values for circRNAs. **(C)** Heatmap showing the hierarchical clustering of differentially expressed circRNAs. Vertical columns represent different tissues, with a bar plot of log_2_ (RPM). Horizontal rows represent circRNAs. Blue to red represents low to high expression. **(D)** Column chart of differentially expressed circRNAs in each comparison. The numbers on column show the quantity of up-regulated (red) and down-regulated (green) circRNAs.

It has been confirmed that circRNAs possess tissue-specific expression characteristics 52-54. To screen dysregulated circRNAs in HBV-related liver cancer, we analyzed circRNA expression profiles using the DEseq software between liver caner tissues and matched pericancerous tissues. The expression abundance of each circRNA was calculated based on RPM (mapped back-splicing junction reads per million mapped reads). The average RPM value of a specific circRNA from three biological replicates was defined as the relative expression of this circRNA for further analysis. The average RPM value was calculated for each circRNA in the tissues from patients with HBV-related liver cancer (Figure 3A). The similar distributions for the dataset of the circRNA profiles were presented as box plots following normalization using the R software package. More than 95% circRNAs had a RPM value of less than 0.25 (Figure 3B). Differentially expressed circRNAs were then screened by fold-change filtering (|log_2_(fold change)|>1) and Student's t-testing (P-value<0.05). As a result, 2,996 circRNAs were identified to be differentially expressed in liver cancer tissues compared with paired pericancerous tissues. The heatmap of inter-sample correlation demonstrated that there was an obvious distinction in circRNA expression patterns between liver cancer tissues and their adjacent normal tissues (Figure 3C). Among the differentially expressed circRNAs, 1,186, 698 and 462 circRNAs were upregulated in liver cancer tissues relative to matched paracancerous tissues, whereas 1,198, 678 and 464 circRNAs were downregulated (Figure 3D). In addition, 502 differentially expressed circRNAs, consisting of 219 upregulated and 283 downregulated circRNAs, were commonly detected in all liver cancer tissues from the patients.

### Functional categorization of differentially expressed circRNAs

**Figure 4:**
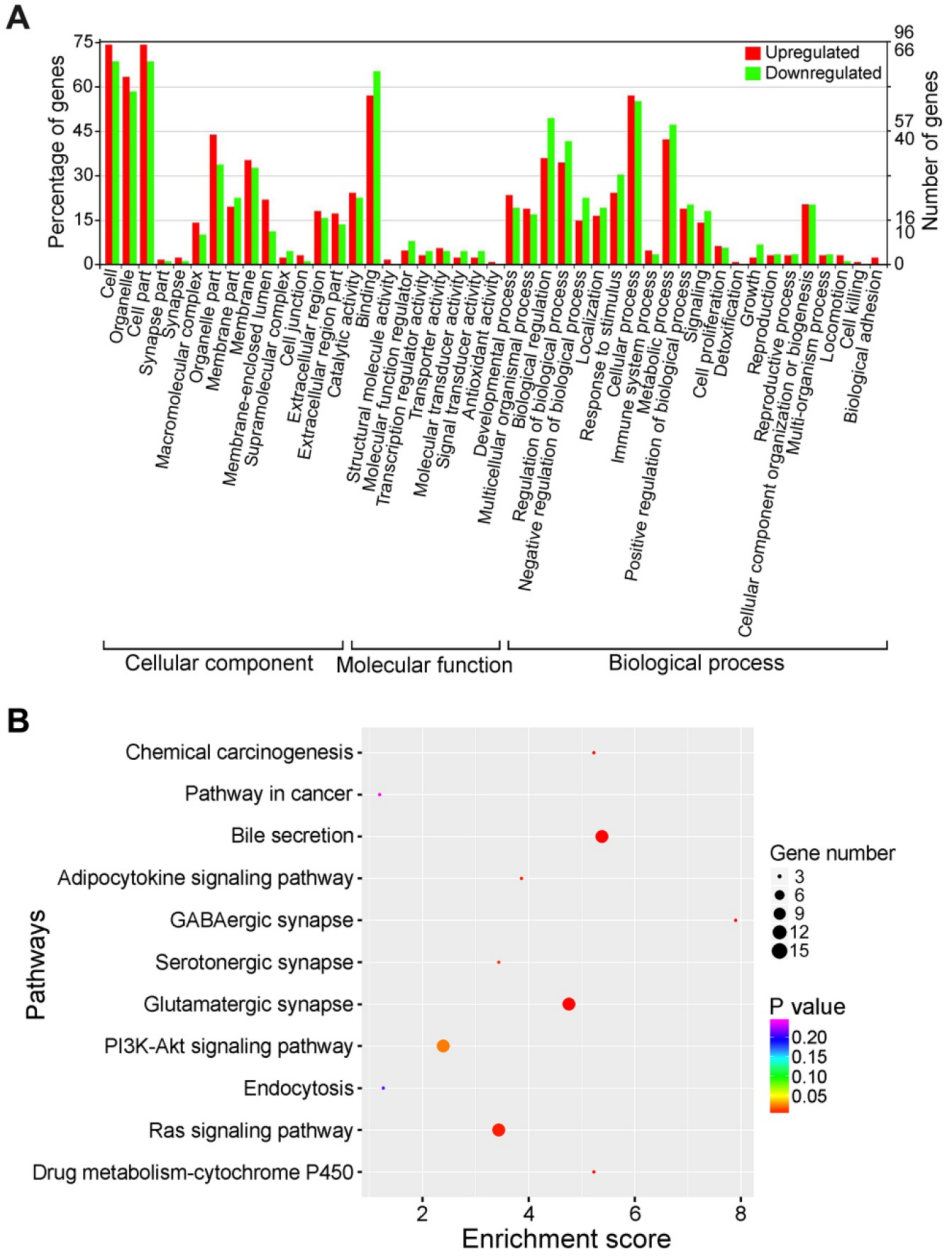
** Function classification of differentially expressed circRNAs. (A)** GO classification of differentially expressed circRNAs. The distributions of gene functions for differentially expressed circRNAs are shown. The functions of circRNA-host genes cover three main categories: cellular component, molecular function and biological process. **(B)** KEGG pathway enrichment of the source genes of differentially expressed circRNAs. The top 10 of pathway enrichments are shown. The y-axis represents KEGG pathways, and the x-axis represents the enrichment score. The size of the points refers to the number of circRNA-host genes mapped to each pathway.

The differential expression of circRNAs suggests potential correlations with their functions. In order to reveal the biological functions of differentially expressed circRNAs, GO assignments were exploited to categorize the functions of the parental genes of dysregulated circRNAs. In the cellular component aspect, the majority of source genes of differentially expressed circRNAs were located within 'cell', 'cell part' and 'organelle' (Figure 4A). 'Binding' and 'cellular process' were the top enriched molecular function and biological process terms, respectively. Specially, several genes were classified into 'cell proliferation', 'growth' and 'cell killing' under the biological process category. These findings implied that these aberrantly expressed circRNAs were engaged in the growth and death of liver cancer cells. Since host immune system exerts a key function in the recognition and elimination of tumor cells 55, the differentially expressed circRNAs involved in 'immune system process' might function in the biological processes of cancer progression and metastasis by functioning as immune modulators.

The top 10 KEGG pathway enrichments were presented in Figure 4B. The host genes of differentially expressed circRNAs were associated with 'Chemical carcinogenesis', 'Pathways in cancer', 'Bile secretion', 'Adipocytokine signaling pathway' and 'GABAergic synapse'. Therefore, the differentially expressed circRNAs might be implicated in cancer-related pathways. Collectively, KEGG pathway analysis suggested that circRNAs might function as critical players in the progression of HBV-related liver cancer.

### Construction of the circRNA-miRNA interaction network

**Figure 5:**
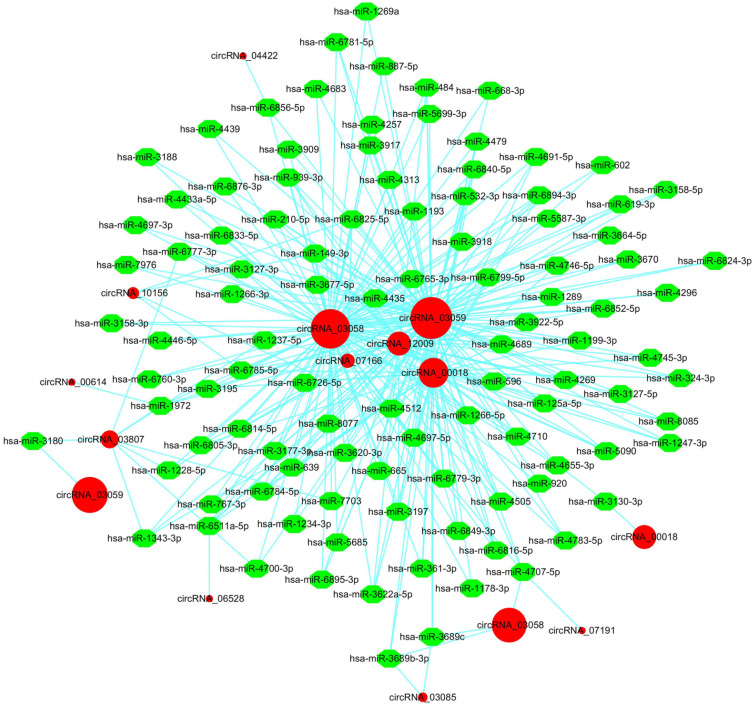
** The circRNA-miRNA interaction network in HBV-related liver cancer.** The top 300 circRNA-miRNA interactions are presented. The network consisting of 12 circRNAs and their target miRNAs was delineated using the Cytoscape software. Red circular nodes represent circRNAs. Green octagon nodes represent miRNAs.

**Figure 6:**
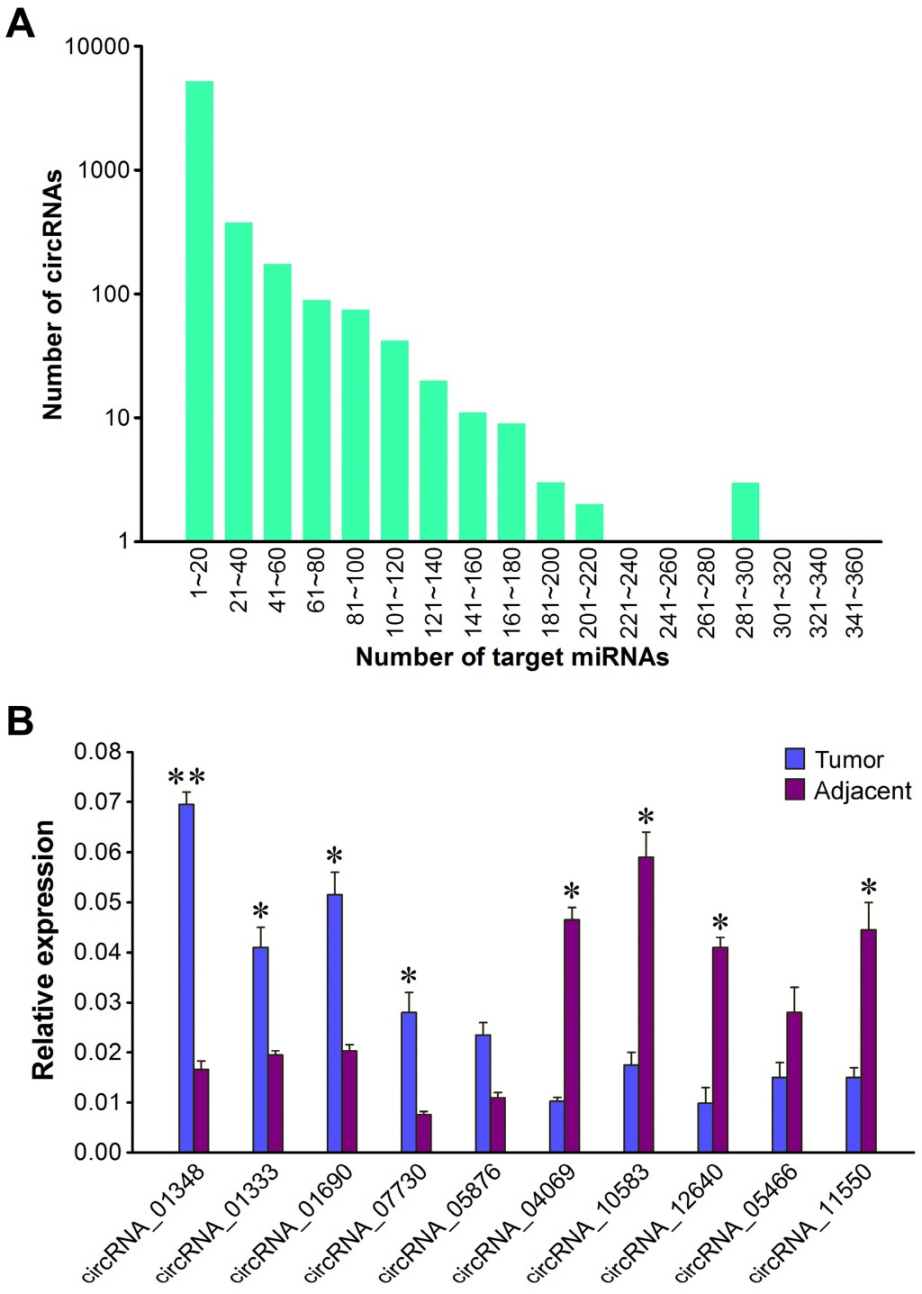
** qRT-PCR validation of differentially expressed circRNAs. (A)** Number of miRNA-binding sites per circRNA. **(B)** Validation of ten differentially expressed circRNAs by qRT-PCR assay. The expression of *GAPDH* gene was used as the internal reference. The values were means ± SD. Statistical analysis was performed with student *t*-test; *P<0.05, **P<0.01.

It is widely accepted that circRNAs can offset miRNA-mediated gene regulation by acting as miRNA sponges 56. For instance, the ciRS-7/Cdr1as circRNA contained over 70 miR-7 binding sites, while Sry circRNA harbored 16 miR-138 binding sites, both of which could modulate gene expression by functioning as miRNA sponges 17. Hence, we identified potential target miRNAs of liver cancer circRNAs using the miRanda software 41. The predicted circRNA-miRNA interaction network was generated by Cytoscape 42. In total, 6,020 (45.87%) circRNAs harbored putative binding sites for 1,654 miRNAs (data not shown). To clearly visualize the intricate interaction between circRNAs and their target miRNAs, we chose the top 300 circRNA-miRNA interaction with significant P-values to map a network. In the circRNA-miRNA interaction network, 106 miRNAs combining with 12 circRNAs were among the top 300 relationships (Figure 5). The result showed that a single circRNA could serve as an antagonist of multiple miRNAs. For example, circRNA_03058 and circRNA_03059 were predicted to target more than 100 miRNAs. Particularly, both of them possessed putative binding sites for hsa-miR-3664-5p, hsa-miR-4296 and hsa-miR-639. Different circRNAs harboring binding sites for the same miRNA might corporately soak up this miRNA and thus modulate its target gene expression in HBV-related liver cancer. Oppositely, circRNA_04422, circRNA_00614, circRNA_06528 and circRNA_07191 acted as molecular sponges for only one miRNA. We conducted a detailed analysis of the miRNA binding sites in the 6,020 circRNAs. The result indicated that the majority of circRNAs (5,209, 86.53%) harbored 1-20 miRNA binding sites (Figure 6A). A total of 801 circRNAs (13.30%) could target 21-200 miRNAs. Ten circRNAs (0.17%) were capable of binding to over 200 miRNAs. Thus, the circRNA-miRNA axes might form a complex regulatory network in gene expression.

### Validation of differentially expressed circRNAs in HBV-related liver cancer

In order to validate the expression difference of ten randomly selected circRNAs revealed by Illumina sequencing, we designed divergent primers for qRT-PCR quantification of RNA samples from each tumor sample and its paired adjacent normal tissue. The results showed that all the ten circRNAs could be detected in both liver cancer tissues and matched pericancerous tissues (Figure 6B). Of the validated circRNAs, five (circRNA_01348, circRNA_01333, circRNA_01690, circRNA_07730 and circRNA_05876) were remarkably upregulated in liver cancer tissues relative to matched pericancerous tissues, while five (circRNA_04069, circRNA_10583, circRNA_12640, circRNA_05466 and circRNA_11550) were more highly expressed in paired pericancerous tissues. The qRT-PCR results, in accordance with circRNA sequencing data, verified that these circRNAs displayed distinct expression patterns between liver cancer tissues and matched pericancerous tissues. These results also showed the accuracy and reliability of the high-throughput RNA sequencing data.

### Determination for the modulatory role of the circRNA_10156/miR-149-3p/Akt1 axis in liver cancer

**Figure 7:**
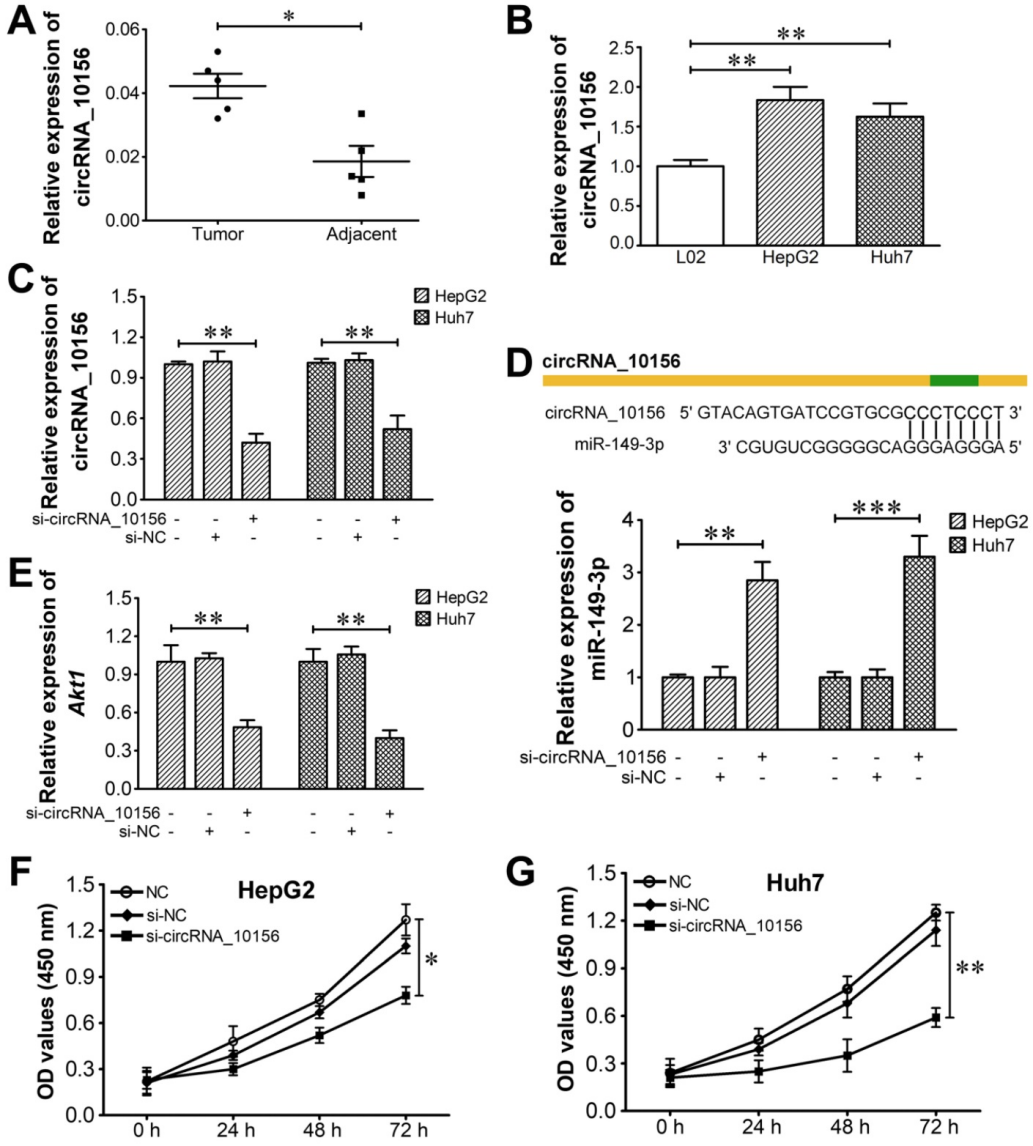
** CircRNA_10156 promotes liver cancer cell proliferation via the miR-149-3p/Akt1 pathway. (A)** The expression of circRNA_10156 in five paired tumor and non-tumorous tissues was detected using qRT-PCR. **(B)** The expression of circRNA_10156 in liver cancer cell lines (HepG2 and Huh7) and the normal hepatic cell line (L02) was examined.** (C)** CircRNA_10156 knockdown in HepG2 and Huh7 cells was achieved by transfection of si-circRNA_10156, as confirmed using qRT-PCR. Cells treated with transfection reagent alone were used as negative controls. **(D)** CircRNA_10156 knockdown upregulates the expression of miR-149-3p in liver cancer cell lines. The miR-149-3p binding site in circRNA_10156 predicted by miRanda is shown. **(E)** CircRNA_10156 knockdown reduces the mRNA level of *Akt1* in liver cancer cell lines. Effect of circRNA_10156 knockdown on the proliferation of HepG2 **(F)** and Huh7 cells **(G)** was detected by CCK-8 assay. *P<0.05, **P<0.01, ***P<0.001.

The identified circRNAs were blasted against circBase, a database of known human circRNAs (http://www.circbase.org/), to determine if they have been reported. CircRNA_10156 was identified as a circRNA in circBase (circBase ID: hsa_circ_0035431). CircRNA_10156 locates at chr15: 57730182-57734676 and its associated gene symbol is CGNL1. It is derived from the *CGNL1* gene exons 3-5, with a spliced mature sequence length of 1,818 bp. It was reported that CGNL1, an endothelial junction complex protein, was capable of regulating vascular growth 57. CircRNA_10156 was predicted to be abundantly expressed in liver cancer tissues relative to corresponding adjacent normal tissues. Based on these clues, we finally determined circRNA_10156 as a target molecule to investigate the possible mechanism of circRNAs in liver cancer pathogenesis. In order to disclose the biological function of circRNA_10156 in liver cancer, we predicted the potential target of circRNA_10156 by adopting the miRanda algorithm. It turned out that circRNA_10156 might work as a molecular sponge for miR-149-3p (Figure 5), which plays an important role in modulating the Akt1 signaling pathway 58-60. Thus, we speculated that circRNA_10156 might be involved in liver cancer progression. We first detected the expression level of circRNA_10156 in five pairs of liver cancer tissues by qRT-PCR. As expected, the expression of circRNA_10156 was obviously higher in liver cancer tissues than that in matched paracancerous tissues (P<0.05; Figure 7A). Likewise, circRNA_10156 was significantly upregulated in HepG2 and Huh7 cells when compared to human normal liver L02 cells (P<0.01; Figure 7B). We then performed loss-of-functional experiments to verify the biological action of circRNA_10156. Synthesized specific siRNAs (si-circRNA_10156) were used to reduce the expression of circRNA_10156 in HepG2 and Huh7 cells. As shown in Figure 7C, si-circRNA_10156 could significantly lower the expression level of circRNA_10156 in HepG2 and Huh7 cells (P<0.01). miR-149-3p was remarkably upregulated in si-circRNA_10156-transfected HepG2 (P<0.01; Figure 7D) and Huh7 cells (P<0.001) compared with the negative control group, while the mRNA level of* Akt1* was markedly decreased in circRNA_10156-knockdown cells (P<0.01; Figure 7E). CircRNA_10156 knockdown markedly suppressed cell proliferation in both HepG2 (P<0.05; Figure 7F) and Huh7 cells (P<0.01; Figure 7G). Altogether, these results indicated that circRNA_10156 could regulate liver cancer cell proliferation through the miR-149-3p/Akt1 pathway.

## Discussion

Recently, circRNAs have become an important research hotspot in the field of RNA due to their stable structures and high conservation among different species 61. CircRNAs show tissue type-dependent and developmental stage-specific expression manners 62. Remarkably, circRNAs are tightly associated with the occurrence and progression of human malignancies 63. Given its high metastatic and recurrent rates, liver cancer is a primary cause of cancer-related death 64-66. Thus, it is essential to discover novel biomarkers for improved intervention of liver cancer. Previous studies indicated that circRNAs could control liver cancer growth and metastasis 67-69, demonstrating that circRNAs may represent novel biomarkers and therapeutic targets in liver cancer. In the present study, we identified 13,124 unique circRNAs in patients with HBV-related liver cancer, 6,844 (52.15%) of which were matched in circBase. The remaining circRNAs that had not been annotated in circBase might be newly discovered circRNAs. Thus, our data might serve as a valuable resource for the development of novel circRNAs as useful biomarkers or therapeutic targets in liver cancer.

Increasing evidence shows that circRNAs display tissue-dependent expression patterns underlining their possible biological importance. Previously, the abnormally expressed circRNAs have been identified in HBV-related HCC via circRNA microarray detection 33. A total of 226 circRNAs were identified to be dysregulated, of which 189 were remarkably upregulated while 37 were downregulated in HCC tissues. Another research group revealed that 61 circRNAs showed different expression patterns between HCC tissues and paracancerous tissues according to the microarray analysis 70. Additionally, a previous study indicated that 24 and 23 circRNAs were obviously upregulated and downregulated in HCC tissues relative to non-tumorous tissues, according to the microarray analysis 71. Here, we exploited an experimental strategy of the high-throughput RNA sequencing combined with bioinformatics approaches, by which more dysregulated circRNAs (2,996) were discovered in HBV-related liver cancer tissues. Accordingly, our approach may be useful to identify new potential biomarkers for liver cancer.

It is known that deregulated circRNAs hold great promise as important biomarkers for the diagnosis and prognosis of liver cancer. A meta-analysis of the efficacy of aberrantly expressed circRNAs for HCC diagnosis was conducted 72. It seemed that the diagnostic performance of upregulated circRNAs for HCC diagnosis was superior to that of downregulated circRNAs. Huang et al. 73 also performed a meta-analysis of the diagnostic and prognostic values of circRNAs in HCC. They found that high expression of oncogenic circRNAs tightly correlated with poor clinicopathological features and were relevant to poor overall survival of HCC patients. In terms of both diagnostic and prognostic values, tumor-suppressive circRNAs proved the contrary. More importantly, these circRNAs showed good sensitivity and specificity in differentiating HCC from controls. In the present study, 219 upregulated and 283 downregulated circRNAs were commonly detected in all liver cancer tissues, respectively. These circRNAs might serve as potential diagnostic and prognostic indicators of liver cancer. However, their association with clinicopathological features of liver cancer patients deserves further exploration. More work is needed to detect the sensitivity and specificity of these circRNAs-based diagnostics. It is essential to elucidate the genuine function of differentially expressed circRNAs identified in this study during liver cancer progression. The characterization of oncogenic or tumor-suppressive circRNAs will provide new insight for circRNA-based diagnostic or prognostic strategies. Collectively, more clinical studies in large cohorts are demanded to verify the clinical significance of liver cancer circRNAs.

Given that miRNAs have important roles in cancer biology, circRNAs are widely involved in cancer development through their regulatory effects on various miRNAs. The pre-existing studies have suggested that circRNAs work as important participants in the carcinogenesis and progression of liver cancer by acting as miRNA sponges. For instance, hsa_circ_0005986 was lowly expressed in HCC compared with adjacent non-cancerous tissues 74. Also, its expression level was associated with clinicopathological features and disease progression in HCC patients. Hsa_circ_0005986 could restrain HCC cell proliferation by sponging miR-129-5p to increase Notch1 expression. These findings suggested that hsa_circ_0005986 might represent a new biomarker for HCC. In contrast, hsa_circ_101280 was reported to be upregulated in HCC 75. It favored proliferation and inhibited apoptosis of HCC cells by targeting miR-375 and upregulating Janus kinase 2 (JAK2). We speculated that aberrantly expressed circRNAs might regulate liver cancer progression through the similar mechanism. CircRNA_10156 was significantly upregulated and we focused on its biological function in liver cancer. Further study showed that circRNA_10156 could promote liver cancer progression by acting as a molecular sponge for miR-149-3p. CircRNA_10156 might represent a novel therapeutic target in liver cancer. Remarkably, the role of miR-149-3p in cancer progression has been extensively studied. Bellazzo et al. 76 identified miR-149-3p as a negative regulator of the tumor suppressor DAB2IP. Moreover, miR-149-3p could promote cancer cell growth, invasiveness and dissemination by inhibiting DAB2IP expression in both cancer and stromal cells. miR-149-3p could also directly interact with killin (KLLN), which was shown to restrain cancer cell growth by increasing p53 expression 77,78. Long intergenic non-protein-coding RNA 472 (LINC00472) could downregulate miR-149-3p and miR-4270 to elevate KLLN expression and activate the p53 signaling pathway, thereby suppressing the proliferation, invasion and migration of non-small-cell lung cancer (NSCLC) cells 77. In addition, miR-149-3p was able to lower the expression of polo-like kinase 1 (PLK1) by targeting the 3' untranslated region (UTR) of *PLK1* mRNA 79. PLK1 acts as an important regulator of apoptosis and cell cycle progression. Accordingly, miR-149-3p-mediated downregulation of PLK1 resulted in the suppression of clonogenicity and the induction of apoptotic cell death in HeLa cells. Collectively, miR-149-3p plays a double-edged sword role by exerting both pro- and anti-tumorigenic effects during cancer development. miR-149-3p can negatively regulate the expression of multiple genes that take part in cancer pathogenesis. We preliminarily verified the role of the circRNA_10156/miR-149-3p/*Ak1* regulatory axis in liver cancer. Nevertheless, other signaling pathways may also mediate the regulatory role of circRNA_10156/miR-149-3p in liver cancer. Thus, it is essential to elucidate the detailed mechanisms associated with the contribution of circRNA_10156 to liver cancer progression in future studies.

## Conclusion

This study provided a landscape of dysregulated circRNAs that might be valuable for the diagnosis and therapy of liver cancer. Moreover, these aberrantly expressed circRNAs might be involved in the pathogenesis of HBV-associated liver cancer by regulating cancer-related molecules or pathways. Further study on the functional role of these circRNAs will increase our understanding of the molecular mechanisms relevant to the initiation and progression of liver cancer. Additionally, our results highlighted the possibility that circRNA_10156 might be used as a promising therapeutic target for liver cancer intervention. These findings not only help to comprehensively uncover the functional roles of circRNAs in HBV-related liver cancer, but also provide promising therapeutic targets for improving the outcomes in patients with HBV-related liver cancer. This study may lay the foundation for further explorations on the biological function, diagnostic and therapeutic potentials of circRNAs in cancer.

## Supplementary Material

Supplementary figures and tables.

## Figures and Tables

**Table 1:** Clinical and pathological characteristics of HBV-related liver cancer patients

Patient No.	Age	Gender	Stage	Grade
Case 1	53	Male	T2	G2
Case 2	69	Male	T2	G2
Case 3	47	Male	T2	G2
Case 4	56	Male	T2	G2
Case 5	51	Female	T2	G2

**Table 2:** General features of the RNA sequencing data

Sample	Case 1		Case 2		Case 3	
	Tumor	Adjacent	Tumor	Adjacent	Tumor	Adjacent
Raw reads (million)	74.95	83.70	78.65	86.62	84.78	88.12
Clean reads (million)	68.4	76.12	71.29	77.74	75.08	79.95
Clean bases	10.21 G	11.36 G	10.65 G	11.61 G	11.2 G	11.94 G
Valid bases (%)	90.85	90.49	90.24	89.32	88.05	90.31
Q30 (%)	97.1	97.04	97.18	97.02	96.8	97.25
GC content (%)	55.81	54.16	55.22	57.41	55.54	54.66

Phred-like quality scores (Q-scores) were used to assess the accuracy of nucleotide identity data from a sequencing run. Q30 represents the probability of an incorrect base call of 1 in 1000.
