# Supplementary material for: Circular RNA Expression Profiles and the Pro-tumorigenic Function of CircRNA_10156 in Hepatitis B Virus-Related Liver Cancer
Source: Int J Med Sci. 2020 May 30;17(10):1351–65. doi: 10.7150/ijms.45637 (PMC7330659; doi:10.7150/ijms.45637)
Supplement: Supplementary file 1 — Supplementary figures and tables. [file ijmsv17p1351s1.pdf]

Supplementary materials

Circular RNA Expression Profiles and the Pro-tumorigenic Function of CircRNA_10156 in Hepatitis B Virus-Related Liver Cancer

Man Wang, Bianli Gu, Guoliang Yao, Peifeng Li, Kun Wang

Table S1. The primer sequences used for qRT-PCR assay in this study

CircRNA ID	Primer sequences (5'-3')
circRNA_01348	Forward: TTTTTGGTGAATTCTGTTTGG
	Reverse: ATCAACTGCCCGAATTCCTT
circRNA_01333	Forward: CTTGGCTGAGATGGTGATAGC
	Reverse: TCAGCTCTTTTCAGCATTTCC
circRNA_01690	Forward: TTTGCTACTGCACCAACAGG
	Reverse: CTTTTCAGCTCCTGGGGTTT
circRNA_07730	Forward: TTGGCTCTTCCTCAATGCTT
	Reverse: GACAAGATTCCGTCTTCATGC
circRNA_05876	Forward: GCATTTTTACGGACCCAATC
	Reverse: TACACTCCCCATTGCTCCTC
circRNA_04069	Forward: GCTTGAGTGCTCAGCTGTTCT
	Reverse: CCCAACAGTCCTCCACAGTC

circRNA_10583	Forward: GCCAGGTGCTCCTAAGAAAG
	Reverse: GCTGAATTTGAACCTTCACACA
circRNA_12640	Forward: CTTGAGCGCCCTCTACTCAG
	Reverse: GATCTTCTGGGTGCTGTCGT
circRNA_05466	Forward: TGTGGCAGAATTGGTTGGTA
	Reverse: AGTGGTTCCCCAGCTCTTTT
circRNA_11550	Forward: TGATGGGTATTTGGGCATTT
	Reverse: GGGCATATGGGAGGGATAGT
circRNA_10156	Forward: GGTCTTTGAGAAAATCCAGACC
	Reverse: TTCCTGCTGCACATGTTGAT
miR-149-3p	Forward: GGTCTTTGAGAAAATCCAGACC
	Reverse: TTCCTGCTGCACATGTTGAT
Akt1	Forward: TCCCGAGGCCAAGTCCTT
	Reverse: CCGCCAAGCCTCTGCTT
GAPDH	Forward: GAAGGTGAAGGTCGGAGTC
	Reverse: GAAGATGGTGATGGGATTTC
U6	Forward: CTCGCTTCGGCAGCACATATACT
	Reverse: ACGCTTCACGAATTTGCGTGTC

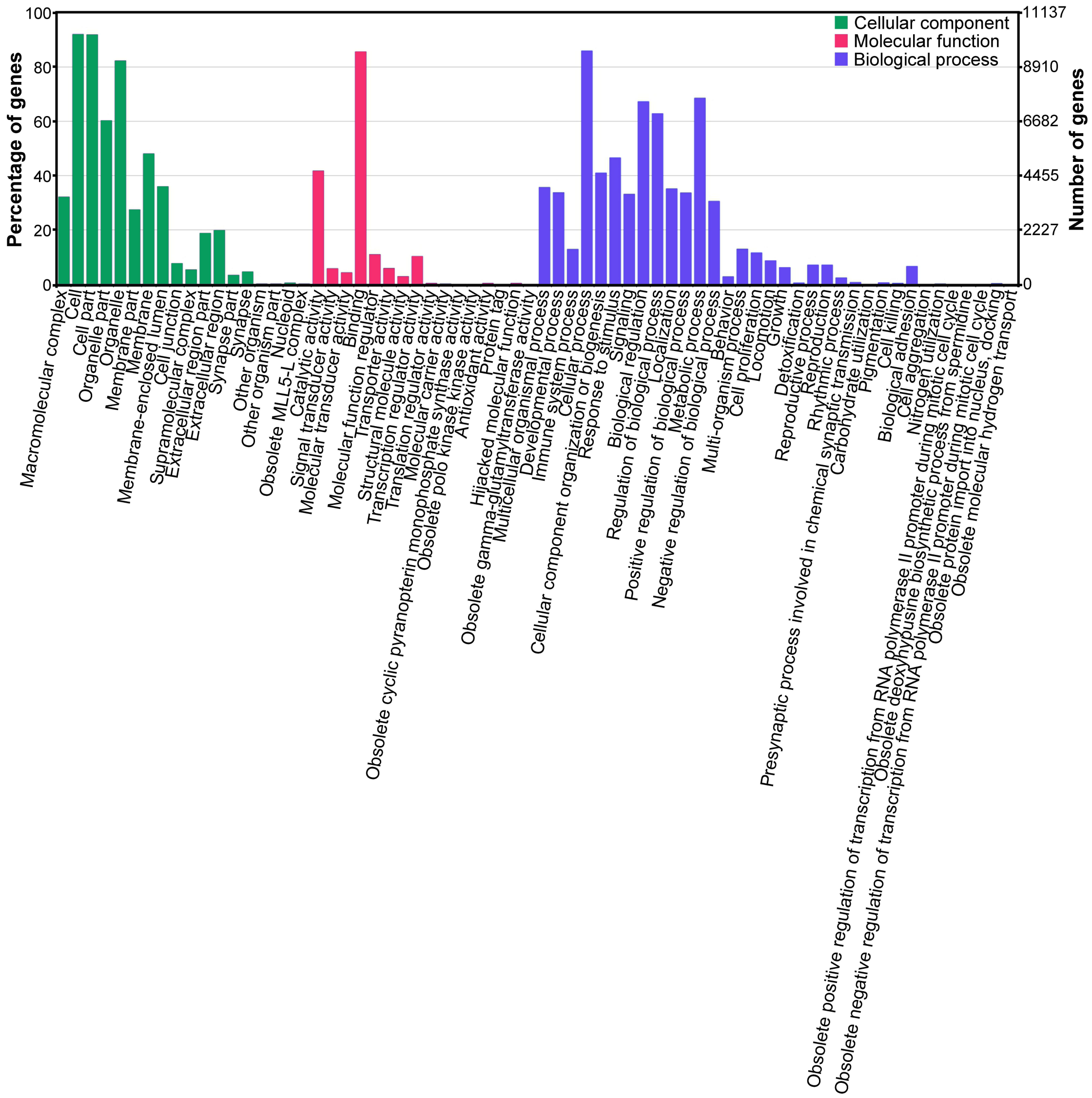

Figure S1. Histogram presentations of GO classification of circRNA-host genes. A total of 11,724 (89.33%) circRNA-host genes were classified into three major functional categories (cellular component, molecular function and biological process) and 68 subcategories. The x-axis indicates different GO terms; left y-axis indicates the percentage of genes in a category; right y-axis indicates the number of genes in each category.

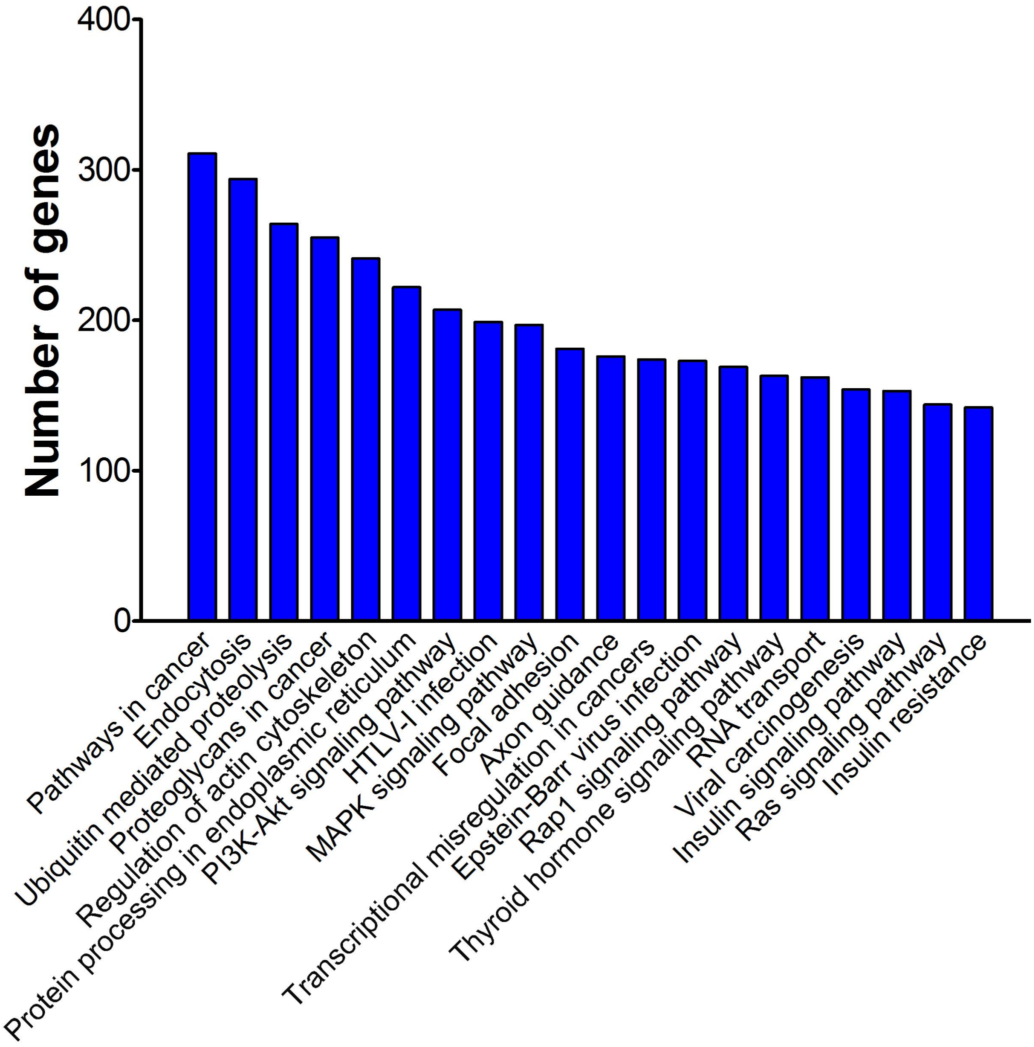

Figure S2. KEGG functional analysis of circRNA-host genes. The x-axis represents KEGG terms, and the y-axis represents the number of circRNAs. The top 20 KEGG pathways are presented.
